# Complete Genome Sequence of *Mycoplasma suis* and Insights into Its Biology and Adaption to an Erythrocyte Niche

**DOI:** 10.1371/journal.pone.0019574

**Published:** 2011-05-10

**Authors:** Ana M. S. Guimaraes, Andrea P. Santos, Phillip SanMiguel, Thomas Walter, Jorge Timenetsky, Joanne B. Messick

**Affiliations:** 1 Department of Comparative Pathobiology, School of Veterinary Medicine, Purdue University, West Lafayette, Indiana, United States of America; 2 CAPES-Fulbright Program, Ministério da Educação, Brasília, Brazil; 3 Purdue Genomics Core Facility, Purdue University, West Lafayette, Indiana, United States of America; 4 Department of Biological Sciences, Purdue University, West Lafayette, Indiana, United States of America; 5 Departamento de Microbiologia, Instituto de Ciencias Biomedicas, Universidade de Sao Paulo, Sao Paulo, Brazil; Virginia Polytechnic Institute and State University, United States of America

## Abstract

*Mycoplasma suis*, the causative agent of porcine infectious anemia, has never been cultured *in vitro* and mechanisms by which it causes disease are poorly understood. Thus, the objective herein was to use whole genome sequencing and analysis of *M. suis* to define pathogenicity mechanisms and biochemical pathways. *M. suis* was harvested from the blood of an experimentally infected pig. Following DNA extraction and construction of a paired end library, whole-genome sequencing was performed using GS-FLX (454) and Titanium chemistry. Reads on paired-end constructs were assembled using GS De Novo Assembler and gaps closed by primer walking; assembly was validated by PFGE. Glimmer and Manatee Annotation Engine were used to predict and annotate protein-coding sequences (CDS). The *M. suis* genome consists of a single, 742,431 bp chromosome with low G+C content of 31.1%. A total of 844 CDS, 3 single copies, unlinked rRNA genes and 32 tRNAs were identified. Gene homologies and GC skew graph show that *M. suis* has a typical *Mollicutes oriC*. The predicted metabolic pathway is concise, showing evidence of adaptation to blood environment. *M. suis* is a glycolytic species, obtaining energy through sugars fermentation and ATP-synthase. The pentose-phosphate pathway, metabolism of cofactors and vitamins, pyruvate dehydrogenase and NAD^+^ kinase are missing. Thus, ribose, NADH, NADPH and coenzyme A are possibly essential for its growth. *M. suis* can generate purines from hypoxanthine, which is secreted by RBCs, and cytidine nucleotides from uracil. Toxins orthologs were not identified. We suggest that *M. suis* may cause disease by scavenging and competing for host' nutrients, leading to decreased life-span of RBCs. In summary, genome analysis shows that *M. suis* is dependent on host cell metabolism and this characteristic is likely to be linked to its pathogenicity. The prediction of essential nutrients will aid the development of *in vitro* cultivation systems.

## Introduction

*Eperythrozoon* and *Haemobartonella* organisms were first described in Germany in the 1920's [1], [2]. Their small size, erythrocyte parasitism, gram-negative staining and putative arthropod transmission led to their initial classification within the *Anaplasmataceae* family, *Ricketsialles* order [3]. However, in 1997 the 16S risobomal RNA (rRNA) gene sequences of some of these organisms (*Eperythrozoon suis*, *Eperythrozoon wenyonii*, *Haemobartonella muris* and *Haemobartonella felis*) were sequenced and phylogenetically analyzed [4], [5]. Surprisingly, these bacteria showed close homology to members of the *Mycoplasma* genus, in the *Mycoplasmataceae* family, *Mollicute* class. An official proposal for reclassification of these four organisms was published in 2001 [6] and subsequent analyses of other *Haemobartonella* and *Eperythrozoon* organisms revealed that these organisms were also members of the *Mycoplasma* genus [7]. Since their taxonomic change, several novel hemotropic mycoplasmas (hemoplasmas) species have been detected in various mammals and they comprise a distinctive group within a genus that was previously known only as mucosal or joint parasites [8].

The causative agent of the porcine infectious anemia (Eperythrozoonosis), *Eperythrozoon suis*, was initially described in the early 1930's in the United States, but in 2001, it was reclassified as *Mycoplasma suis* [6], [9]. This organism has been described in domestic pigs in South and North America, Europe and Asia [10]–[12, Guimaraes et al., unpublished data]. Pathogenicity studies involving this bacterium have been hampered mainly due to an inability to cultivate the hemoplasmas *in vitro*. Observations from field and experimental infection of pigs suggest that there are two forms of disease: an acute stage with severe hemolytic anemia or a chronic form with poorly defined clinical signs. Both disease forms have been reported on commercial pig farms [10], [13]–[16]. Chronically infected animals, however, are more commonly seen, possible due to the addition of subtherapeutic levels of tetracycline in the feed. Reproductive problems in sows and reduced weight gain in feeder pigs have been associated with this form of infection [13]–[16]. The acute disease is mainly observed in suckling pigs and in animals suffering from an immunosuppressive event [17], [18]. The acute form of the disease cannot be experimentally induced in healthy pigs unless they are immunosuppressed or splenectomized. However, splenectomy of a chronically infected pig will lead to an acute, fulminating disease.

Although *M. suis* was recognized as a pathogen of pigs over 80 years ago, little has been done to better understand its virulence and mechanisms of pathogenicity, nor to understand what factors are needed for its survival. Cellular adhesins that mediates the specific attachment *M. suis* to swine red blood cells and the molecular basis of adherence has not been fully studied. In addition, there are no reports detailing the surface architecture of *M. suis*. This study was undertaken as the first step toward understanding the mechanisms by which *M. suis* causes disease and establishes chronic infection, as well as to gain new insights into the biochemical pathways that permit its survival and growth *in vitro*. Herein, we present the whole genome sequence of *M. suis* strain Illinois and its computational analysis.

## Results and Discussion

### General genome features

**Figure 1:**
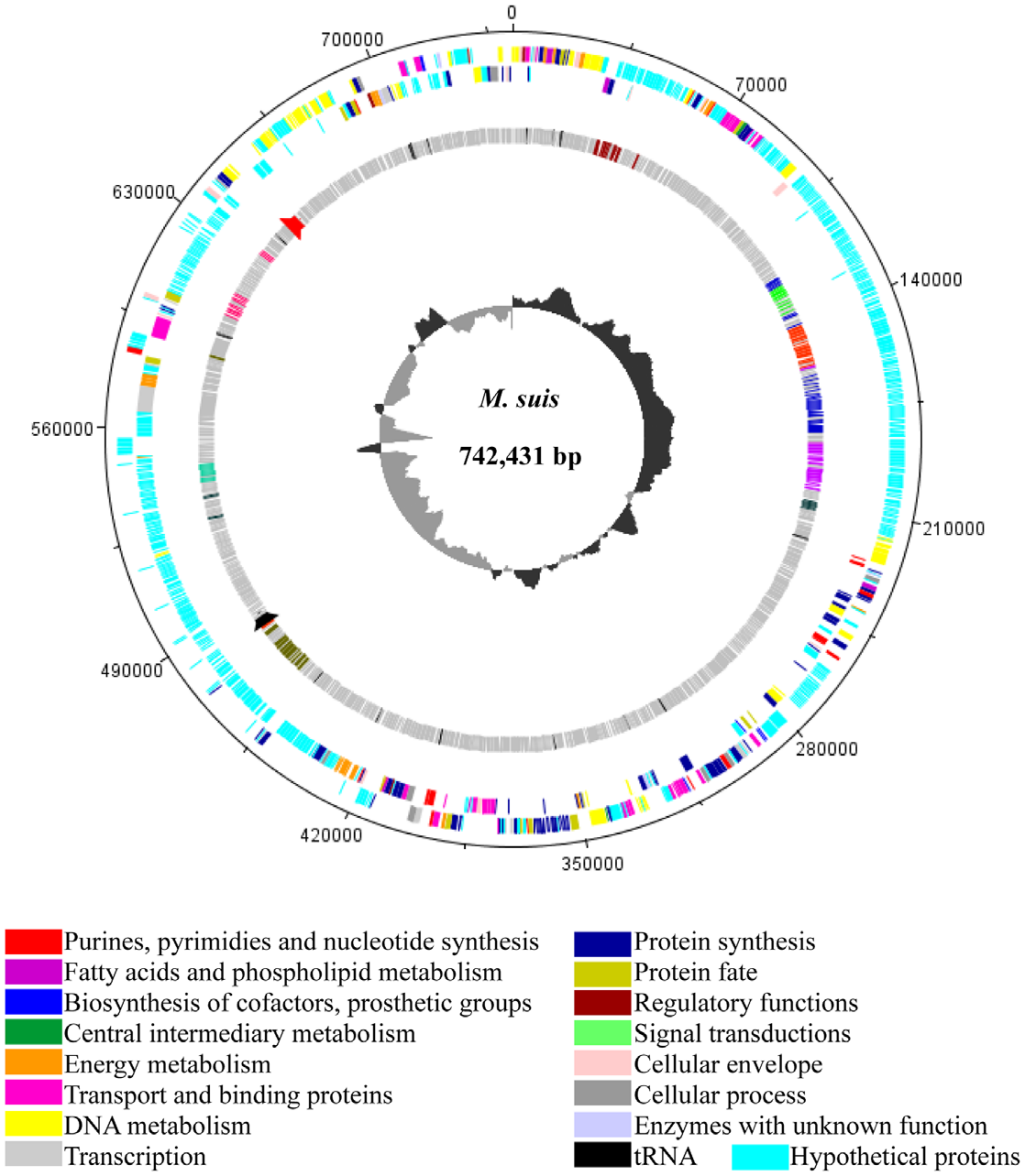
Circular diagram of functional assignments of the *Mycoplasma suis* genome. Starting from the outside, the first circle shows CDSs in the positive strand. Second circle shows CDSs in the negative strand. The third circle shows the 9 largest hypothetical paralogous gene families; colors indicate relationships and do not correlate to functional assignments. Black and red tick marks on the third circle represent the 16S rRNA gene and the 23S/5S rRNA gene operon, respectively. The fourth circle shows the GC skew ([G−C]/[G+C]). The *dnaA* gene is at position 1. Functional assignments are related to TIGR roles. The figure was generated using DNAPlotter version 1.3 from Artemis 12.0, Sanger Institute.

The general genome features of the *M. suis* strain Illinois are shown in Table 1 and Figure 1. Its genome consists of a single, circular chromosome of 742,431 bp with an overall G+C content of 31.1 mol%. A total of 3 ribosomal RNA (rRNA) genes and 32 transfer RNAs (tRNA) were identified. The rRNA genes were found in single copies and unlinked; the 23S and the 5S are organized as an operon and the 16S rRNA is located 166 kb downstream of these genes. This same organization was described only in *Mycoplasma gallisepticum*; however, an additional operon containing all three rRNA genes was also identified [19]. Thus, so far, the presence of a single 16S rRNA gene unlinked to the other rRNAs is a unique feature of *M. suis*.

**Table 1:** General features of *Mycoplasma suis* genome compared to members of pneumoniae, hominis and mycoides phylogenetic groups of Mycoplasma, and Acholeplasma and Phytoplasma.

	Pneumoniae group					Hominis group				Mycoides group	Anaeroplasma and Phytoplasma groups	
Parameter	Msu	Mpn	Mgal-R_l_	Mge	Upa	Mho	Mhy-J	Msy	Mpu	Mmy	Ala	Pas*
Genome (bp)	742,431	816,394	1,012,800	580,076	751,719	665,445	897,405	799,476	963,879	1,211,703	1,496,992	860,631
G+C content (%)	31.1	40	31	31.7	25.5	27.1	28	28	26.6	24	31	28
CDS	844	677	742	475	613	537	679	694	782	985	1,380	754
Gene density (%)	89.9%	88.7	91	90	93	89.8	88	91	91.4	83	90	73
Average CDS length (bp)	783	1011	1,206	1,040	1,116	1,107	1,178	1,058	1,115	982	981	785
CDS with predicted function	293 (34.7%)	333 (49%)	469 (63%)	323 (68%)	325(53%)	345 (64%)	412 (60%)	464 (67%)	486 (62%)	581 (59%)	1,006 (73%)	446 (59%)
Conserved hypothetical CDS	34 (4.0%)	181 (27%)	150 (20%)	56 (12%)	116 (19%)	86 (16%)	109 (16%)	167 (24%)	92 (12%)	138 (14%)	NR	51 (7%)
Hypothetical CDS	517 (61.3%)	163 (24%)	123 (17%)	96 (20%)	172 (28%)	106 (20%)	158 (24%)	63 (9%)	204 (26%)	266 (27%)	NR	257 (34%)
No. of tRNAs	32	39	33	40	33	33	30	34	29	30	35	32
No. of rRNAs												
16S	1	1	2	1	2	2	1	2	1	2	2	2
23S	1	1	2	1	2	2	1	2	1	2	2	2
5S	1	1	2	1	2	2	1	3	1	2	2	2

*‘*Candidatus* Phytoplasma asteris’ has two plasmids, EcOYM with 5,025 bp and pOYM with 3,932 bp. Msu = *M. suis*, Mpn = *M. pneumoniae*, Mgal-R_l_ = *M. galliseptcium* strain R-low, Mge = *M. genitalium*, Upa = *Ureaplasma parvum* serovar 3, Mho = *M. hominis*, Mhy-J = *M. hyopneumoniae* strain J, Msy = *M. synoviae*, Mpu = *M. pulmonis*, Mmy = *M. mycoides* subsp mycoides, Ala = *Acholeplasma laidlawii*, Pas = ‘*Candidatus* Phytoplasma asteris’. NR = not reported.

The 32 identified tRNAS cover all 20 amino acids and it is within the range for numbers reported in the 41 available *Mollicutes* genomes in NCBI (from 28 to 38 tRNAs). As described for other mycoplasmas, the UGA in the *M. suis* genome is likely to be used as a tryptophan codon; however, a tRNA for selenocysteine [20], which also uses the ‘stop’ codon UGA, was identified. This Sec-tRNA has been described in 6 other mycoplasmas (*M. genitalium*, *M. gallisepticum*, *M. capricolum*, *M. hyorhinis*, *M. crocodyli*, *M. alligatoris*), however its use versus the Trp-tRNA is still unclear.

### Coding DNA sequences (CDS)

A total of 844 CDSs were predicted. The genes comprise 89.9% of the genome, which is a percentage comparable to that of other mycoplasmas. However, the average gene length of 783 bp for *M. suis* is comparable only to Phytoplasma organisms and considerably less than that reported for other mycoplasmas (Table 1). The gene length is also similar to some ricketsia, such as *Anaplasma phagocytophilum* (775 bp), *Neoricketssia sennetsu* (804 bp), *Ehrlichia chaffensis* (840 bp) and *Wolbachia pipientis* (855 bp) [21]. It is noteworthy to mention that, due to the high specificity given by the long-orfs training of Glimmer, very small CDS (approximately 90–120 nucleotides) with no homologs in the GenBank or paralogs in the *M. suis* genome may be missing. This implies that the average gene length could be even shorter. In the absence of experimental evidence, these CDSs remain to be added.

Putative biological function was assigned to only 293 (34.7%) of the CDS; 517 (61.3%) of the CDS were hypothetical proteins whereas 34 (4.0%) were conserved hypothetical proteins. Of these 34 conserved hypothetical proteins, 17 were unique to *M. suis* and *M. haemofelis*. Remarkably, the proportion of hypothetical proteins in the *M. suis* genome greatly exceeds the proportion of these proteins in other *Mollicutes* (Table 1) and other blood-borne pathogens [21], [22]. This fact highlights the divergence of the hemoplasma genome when compared to other *Mollicutes*. Being a wall-less bacteria in a blood environment, hemotropic mycoplasmas probably developed special mechanisms for survival in this environment, most of which are still unknown.

### Origin of replication

**Figure 2:**
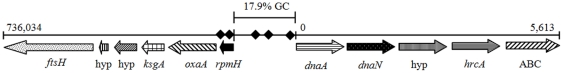
Putative gene organization of the origin of replication of *Mycoplasma suis*. The *dnaA* boxes were initially localized using the Ori Finder software (Gao and Zhang, 2008) and annotated based on the consensus sequence 5′ – TTWTMHAMA – 3′ (Papazisi et al., 2003). One base mismatch was allowed. hyp = hypothetical protein. ⧫ = *dnaA* boxes.

The origin of replication (*ori*C) of only a few *Mollicutes* has been experimentally validated [23]–[26]. These experiments in other bacteria have generally shown that the *ori*C is located in the vicinity of the *dnaA* gene and contains *dnaA* boxes. Thus, this information and the GC-skew graph have been used to predict origins of replication [19], [27], [28]. Assessment of the *ori*Cs in the available mycoplasma genomes reveals variations in their gene organization, but a key signature includes the tandem arrangement of *dnaA* and *dnaN* genes; *M. pneumoniae*, *M. genitalium* and *M. gallisepticum* are the only available genomes in which these genes are not together. Therefore, the *M. suis ori*C was identified based on homologous gene searches, GC-skew graph and *dnaA* box findings. The gene organization of the putative *ori*C is shown in Figure 2. As observed in six other mycoplasmas and in *Escherichia coli*, both *dnaA* and *dnaN* in *M. suis* were found in tandem arrangement and the ribosomal protein L34 (*rpm*H gene) was located in an opposite direction upstream of *dnaA*. The GC-skew graph (Figure 1) shows a significant inversion upstream of the *dnaA* and 6 putative *dnaA* boxes were located near the same gene, providing additional evidence that the origin of replication can be annotated in this region of the genome. In addition, the region between the *dnaA* and the *rpmH* gene comprises 134 nucleotides and its GC content is only 17.9%, which is considered a distinctive characteristic of the *ori*C in prokaryotes [29].

### Metabolic pathways

#### Energy metabolism

The predicted metabolic pathways of *M. suis* are shown in Figure S1 (Table S1 and S2). Mycoplasmas generate ATP by the fermentation of sugars, the oxidation of organic acids (pyruvate and lactate) to acetate plus CO_2_, the hydrolysis of arginine, the proton-translocating ATP synthase, or by combinations of these reactions [30], [31]. *Mycoplasma suis* is a glycolytic species, relying on the generation of energy through fermentation of sugars and ATP-synthase. Accordingly, all enzymes from the Embden-Meyerhoff-Parnas (EMP or glycolysis) pathway as well as F1F0 ATP synthase are present in the genome of *M. suis*. Orthologs to enzymes of the arginine hydrolysis pathway and dihydroxyacetone kinase are absent. Thus, the ATP synthesis through arginine hydrolysis or dephosphorylation of dihydroxyacetone phosphate does not occur in this organism.

Interestingly, the pyruvate metabolism in *M. suis* is incomplete; only an ortholog for lactate dehydrogenase was found. Thus, the production of ATP from the oxidation of pyruvate to acetate is unlikely, given that the pyruvate dehydrogenase complex and the acetate kinase are missing. *Ureaplasma parvum* and *U. urealyticum* genome also do not contain an ortholog for the pyruvate dehydrogenase complex, but activity of this enzyme has been described in *U. urealyticum* [cited in 32]. Although pyruvate dehydrogenase was reported in a proteomic study based on 2D gels and MALDI-TOF analysis of *M. suis* [33], the absence of coenzyme A (CoA) further supports the lack of this enzyme in the genome of *M. suis*. Another possibility is that this organism acquires CoA from its environment. Thus, based on the genome analysis, *M. suis* can only oxidize pyruvate through lactate dehydrogenase, which regenerates NAD^+^. Lactate may represent a possible toxic end-product. It is possible that the balance of reducing power is regulated by the rate of this reaction, which may be higher during anaerobic conditions [30].

#### Pentose phosphate pathway

The pentose phosphate (PP) pathway in mycoplasmas is considered to be incomplete, but functional [34]. However, the whole pathway is apparently absent in *M. suis*. This feature has been described in *Phytoplasma* spp. and is believed to be a result of reductive evolution due to life in an intracellular nutrient-rich environment [35]. Similarly, *M. suis* is also present in a nutrient-rich environment, so that it can acquire metabolites from plasma and red blood cells, and perhaps even intracellularly, as recent evidence suggests [36].

In the absence of a PP pathway, *M. suis* must use alternative mechanisms to generate NADPH. The absence of NAD^+^ kinase also impairs the interconversion of NADH and NAPDH in the cell. At least to partially circumvent this problem, *M. suis* has a non-phosphorylating glyceraldehyde-3-phosphate dehydrogenase (GAPN) (Figure S1). This enzyme is responsible for an irreversible reaction between glyceraldehyde-3-phosphate and 3-phosphoglycerate that reduces NADP to NADPH and has been described in some mycoplasmas and a few other bacterial species [37], [38]. It is believed that GAPN can preserve the production of NADPH in bacteria lacking some of the PPP enzymes [39]. Another explanation for the presence of a GAPN in *M. suis* is that it might be used to maintain glycolysis in conditions of ADP and inorganic phosphate (Pi) depletion and hydrogen peroxide exposure. GAPN is considered more resistant to hydrogen peroxide than the phosphorylating glyceraldehyde-3-phosphate dehydrogenase (GAPDH) [40]. Since reactive oxygen species are continuously produced within red blood cells, oxidative stress to this cell and by extension to *M. suis* is likely high [41]. The use of GAPN may be related to selective pressures imposed on *M. suis* by its dependence on a red cell niche.

The lack of a PP pathway also provides clues for designing a media to support the *in vitro* cultivation of *M. suis*. In the absence of these enzymes, pentoses for DNA and RNA synthesis cannot be produced. In contrast to *M. pneumoniae* [34], ribose is likely to be an essential nutrient for the survival of *M. suis*. This feature would imply that a ribose kinase is present in *M. suis*. Although an ortholog of ribose kinase is missing in mycoplasmas, including the *M. suis*, its activity has been recently described in *M. pneumoniae* [34]. Interestingly, an ortholog of the ribose/galactose importer was not identified; *M. suis* may import this sugar through another ABC-transporter for which the specificity is not yet described.

#### Nucleotide synthesis

Possibly all *Mollicutes* are unable to synthesize *de novo* purine and pyrimidine bases [31], [42] and rather rely on importing them from the environment. Yus et al. [34] determined that adenine and guanine are essential for *M. pneumoniae* growth whereas cytidine was sufficient for pyrimidine synthesis. In *M. suis*, enzymes for the metabolism of inosine monophosphate are present; thus, it is predicted that hypoxanthine scavenged from the environment is sufficient for the synthesis of all purine nucleotides (Figure S1). This is somewhat interesting given the fact that hypoxanthine is essential for *Plasmodium falciparum* growth *in vitro* and the major purine present in the blood of mammals [43]–[45]. Hypoxanthine is secreted as an end product from red blood cell nucleotide metabolism [44], [45], providing a ready source for *M. suis* to import it from the plasma.

For the pyrimidine metabolism of *M. suis*, uracil is likely to provide cytosine nucleotides whereas thymine and/or thymidine are probably needed (the absence of a thymidylate synthase impairs the production of thymine nucleotides from dUMP/uracil). This implies the use of a phosphofructokinase for the conversion of cytidine tirphosphate (CTP) to deoxycytidine diphosphate (CDP) (Figure S1). Some glycolytic enzymes (pyruvate kinase, phosphofructokinase, acetate kinase and phosphoglycerate kinase) have been shown to exert this function in *M. genitalium*, *M. pneumoniae*, *M. fermentans* and *M. capricolum* subsp. *capricolum* [46]. Alternatively, *M. suis* may require CTP or CDP for growth. Finally, the pyruvate kinase present in the genome of *M. suis* is proposed to be used in the conversion of all NDP and dNDP to NTP and dNTP, respectively (Figure S1) [34], [46].

#### Lipid and coenzyme A metabolisms

The lipid metabolism constituted the greatest challenge in the prediction of *M. suis'* metabolic pathways. Similarly, this pathway has been poorly described in mycoplasmas and not all enzymes are identified [34], [47]. The existence of some enzymes is inferred due to the presence of others and the presence of end-products. In the *M. suis* genome, most of the enzymes for the lipid and coenzyme A metabolisms are absent. To circumvent some of the reaction gaps, proteins identified in a *M. suis* proteomic study described elsewhere were used [48]. The result is a similar phospholipid biosynthesis pathway as described for *M. pneumoniae* [34], but still, several enzymes remain to be found and/or identified.

Lipids are major constituents of cell membranes. There are three kinds of membrane lipids: phospholipids, glycolipids and sterol. As described for most mycoplasmas [31], *M. suis* is incapable of fatty acid biosynthesis from acetyl-CoA, an important precursor for membrane lipids. This feature is rare among eubacteria, but is characteristic of a reduced genome and increased dependance on host cell nutrients [31]. Phospholipids and glycolipids are thus believed to be synthesized from glycerol. Although an ortholog for the glycerol uptake facilitator protein (GlpF) is absent, the presence of enzymes scattered throughout the pathway suggests that *M. suis* uses glycerol, as well as fructose, to generate phospholipids (cardiolipin). Given the presence of a choline kinase, it is also possible that phosphatidylcholine is synthesized. The pathway for synthesis of glycolipids appears to be missing; all enzymes involved in the synthesis of glycolipids from phosphatidate and enzymes that provide UDP glucose/galactoase as substrate for these reactions are absent.

In *M. suis*, no orthologs for the seven possible enzymes in CoA metabolism [34] are found. The acyl carrier protein (ACP) and enzymes directly involved in its metabolism (ACP synthase, ACP phosphodiesterase, ACP ligase) are typically found in the available mycoplasma genomes. The absence of ACP and ACP synthase has been described only in *M. hyopneumoniae* (all three sequenced strains). Likewise, *M. hominis* does not have an ACP, but has an ACP synthase, and all other mollicutes, except for *Acholeplasma laidlawii*, ureaplasmas and phytoplasmas, have an ACP phosphodiesterase. Thus, *M. suis* is incapable of using pantothenate for acylation and CoA synthesis. An alternative means of acylation is possibly used in its lipid metabolism.

The described findings suggest that *M. suis* is likely to synthesize phosphatidylglycerol and cardiolipin from exogenous fatty acids and use them as major constituents of the plasma membrane; however, the biosynthetic pathway needs to be thoroughly validated once this organism can be cultured *in vitro*. If gaps cannot be filled by enzymes, yet to be described, the possibility that *M. suis* requires exogenous phospholipids and glycolypids is also plausible. Sterol synthesis does not occur in *M. suis* and thus it is possible, as with other mycoplasmas [49], [50], that this molecule is essential for its growth.

#### Amino acid metabolism

*M. suis* is unable to synthesize any amino acid, which is typical of mycoplasmas. Yus et al. [34] have recently shown that *M. pneumoniae* requires both peptide (peptone) and free amino acids to achieve optimal growth. Since amino acids transporters are few, amino acids need to be supplied in excess to counteract any competition for transporters; peptides can serve as an alternative source of amino acids following intracellular digestion [34]. In the genome of *M. suis* genome only one transporter, an amino acid permease, related to the transport of amino acids, was identified. While the putative transporter for importation of peptides, the *opp* ABC peptide transporter, was not found in the genome of *M. suis*, it is possible that this function is performed by another of the ABC transporters.

#### Vitamin metabolism

Mycoplasmas are not capable of synthesizing vitamins and need to import them from their environment. It is still unclear, however, which vitamins are essential for the growth of these organisms. Although removing vitamins does not greatly affect *in vitro* growth of *M. pneumoniae*, all nine vitamins tested enhanced its growth [34]. Nicotinic acid and riboflavin have been described as essential nutrients for spiroplasma growth [51]. Vitamins from Eagle's minimal essential media (folic acid, nicotinamide, riboflavin, cobalamine) also positively influenced growth of ureaplasmas, but were not essential [52]. Since *M. suis* has ABC transporters for spermine/putrescine and cobalamines, these vitamins are likely to be needed for its survival.

Orthologs for all nine genes involved in folate metabolism are absent in *M. suis*. Further, glycine hydroxymethyltransferase was not found. Thus, it appears that *M. suis* cannot interconvert serine and glycine and must acquire both from the environment. *M. suis* also has an incomplete nicotinate pathway. The enzyme nicotinate phosphoribosyltransferase (PncB), the first committed enzyme in this pathway, has a nonsense mutation followed by a frameshift mutation that may impair or completely ablate its activity. Two additional CDSs related to the NAD metabolism were found: nicotinamide-nucleotide adenylyltransferase (nadD), the second enzyme in the pathway, and a phosphohydrolase having a partial NadD domain and unknown function. Nevertheless, NAD^+^ synthase and NAD^+^ kinase are missing. Interestingly, a NAD^+^ kinase is present in *M. haemofelis* (Santos et al., unpublished). Whether or not *M. suis* can produce NAD^+^/NADH is unknown; however, the absence of NAD^+^ kinase implies that NADPH cannot be generated and thus the organism may get it from external sources.

#### DNA metabolism and repair

At least 28 CDSs in the genome of *M. suis* are likely to be involved in restriction-modification (R-M) systems. One putative type I R-M system (*hsdR*/*hsdM*/*hsdS*) locus was found along with several orphan *hsd* genes scattered throughout the genome. It is unclear if these orphan genes are functional. A type II cytosine-specific methyltransferase, a S-adenosyl-methyltransferase (MraW), a DNA methylase N-4/N-6 domain-containing protein, and a type II restriction enzyme (DpnII) were found in single copies. Among nucleases, endonuclease IV, ribonuclease p, two ribonuclease r and ribonuclease HIII were identified. As expected [53], a thioredoxin reductase system was also found (thioredoxin and thioredoxin reductase).

#### Transporters

Overall, mycoplasmas have fewer transporters than other bacteria [22]. It has been suggested that this reduction has been compensated by using transports with broad substrate specificity [54]. Only 31 (3.65%) of the *M. suis* CDSs comprise transport systems, which is less than that reported for other mycoplasmas (6.8% to 13.4%) [19], [27], [55]. As expected [55], ABC systems-related sequences represent 74.19% (23/31) of all transporter CDSs. The low number of genes devoted to the transport of nutrients indicates two possibilities: these transport systems may have broad substrate specificities and/or other unknown CDSs are involved in transport of biomolecules through the plasma membrane.

### Virulence and pathogenicity mechanisms

The current pathogenesis model for anemia due to hemoplasma infection is based mostly on extravascular hemolysis in the spleen, liver, lungs and bone marrow [18], [56], [57]. It is also believed that an increased osmotic fragility of the erythrocytes caused by hemoplasma attachment may lead to an intravascular hemolysis [58]. There is, however, no genetic evidence provided in this study to support a direct attack on red blood cells leading to its lysis. Thus, no bacterial toxins, orthologs of hemolysins, hemagglutinins or other related sequences could be found in the genome of *M. suis*. It is still possible that an immune mediated destruction of red blood cells may exacerbate the anemia through molecular mimicry and/or exposure of hidden cytoskeletal proteins of erythrocytes [59].

While attachment to the red blood cell may lead to mechanical and/or osmotic damage, the extensive scavenging of nutrients from the host cells by *M. suis* is also likely to play an important role in accelerating the removal of infected cells. A novel model for acute disease due to *M. suis* infection is proposed based on our genetic evidence: *M. suis* competes for and scavenges glucose, inosine, hypoxanthine, amino acids, NADH/NADPH and ribose from the host cell. Thus, the reducing power and energy production (NADH, NADPH, 2,3-DPG, ATP and glutathione disulfide) of the red blood cells is compromised. Consequently, there is increased oxidant stress on the red cell and its life span is shortened, which leads to their premature removal from the circulation and anemia develops. It is still unclear how *M. suis* acquires metabolic compounds from inside the host cell, however invasion of the erythrocyte [36] might aid in this process.

In a search for additional virulence factors of *M. suis*, some adhesins, heat-shock proteins, lipoproteins, protein antigens and proteases were identified. Three orthologs of proteins involved in adhesion were found, including at least two MgPa (DHH family phosphoesterase protein) orthologs and the previously described *M. suis* adhesin G1 [33]. Although one of the putative MgPa proteins has an approximate full length match to known MgPa proteins in the GenBank databases, the other MgPa has 140 additional amino acids at the C-terminal. MgPa proteins have been initially described as part of a tip organelle structure responsible for surface adhesion and possibly motility in *M. genitalium* [60], [61]. However, *M. suis* does not have other proteins that are part of this organelle, such as the accessory proteins of the group Hmw and the MgPa adhesin (adhesin P1). This observation is consistent with electron microscopy analyses of *M. suis*, in which tip-like structures were not observed [62]. While every available mycoplasma seems to have orthologs to MgPa (DHH family phosphoesterase protein), its function in those lacking a tip-structure is unknown.

Heat-shock proteins have been described as virulence factors in prokaryotes [63]. Those identified in *M. suis* include DnaJ, DnaK, and GrpE. DnaK has been previously reported as an immunoreactive protein of *M. suis* [33]. Interestingly, an ortholog for the chaperonin GroEL was not found, however this protein was previously described using 2D gel electhrophoresis and MALDI-TOF analysis of *M. suis* [33]. It is possible that one of the hypothetical proteins of *M. suis* has a similar molecular weight, pI and function, but the protein sequence similarity is not enough to generate significant results. On the other hand, not all *Mollicutes* have GroEL, and perhaps this chaperone also has been lost from the genome of *M. suis*. A role for selective immunologic pressures in the loss of this protein in other bacteria has been suggested [64].

In order to assess possible similarities in virulence factors among mycoplasmas that infect pigs, we compared the CDSs of *M. suis* to known adhesins and antigens of *M. hyopneumoniae* and *M. hyorhinis*. The genomes of three strains of *M. hyopneumoniae* have been completely sequenced, annotated and analyzed (strains J, 7448 and 232), whereas, the sequenced genome of *M. hyorhinis* has not yet been analyzed. There are no available protein sequences of known antigens/adhesins of *M. flocullare* and *M. hyosynoviae*, which are also known to infect pigs. In addition to the MgPa and heat-shock proteins described above, a CDS matching the R2 repetitive motif of the P97 adhesin of *M. hyopneumoniae* was identified in the genome of *M. suis*. This motif is responsible for extracellular matrix binding [65], however it is also repeatedly present in protein KIAA1398 of the pig genome (*Sus scrofa*, accession number 001926148.1); the function of this protein is unknown. A shorter homolog to KIAA1398 in the dog genome (*Canis lupus familiaris*), which contains the same motif, has ribosome binding capabilities and may mediate the interaction between the ribosome and the endoplasmic reticulum membrane [66]. While the relationship between this motif/protein and erythrocyte parasitism is unclear, experimental analysis of this protein is currently underway in our laboratory. Other antigens/adhesins, such as the variable lipoproteins (vlp) of *M. hyorhinis*, were not found in the *M. suis* genome. The lactate dehydrogenase (Ldh), a housekeeping gene known to be specifically immunogenic in *M. hyopneumoniae* [67], is also present in *M. suis*. Whether or not the *M. suis* Ldh is immunogenic, or serum from *M. suis* infected animals cross-react with the *M. hyopneumoniae* Ldh, remains to be elucidated.

The survey for lipoproteins using the LipoP 1.0 algorithm resulted in the identification of only 3 lipoproteins. In addition, orthologs for prolipoprotein diacylglycerol transferase, signal peptidase II, and transacylase were not found in the genome of *M. suis*. This is somewhat surprising given that all mycoplasmas sequenced to date have orthologs to most of these enzymes as well as several lipoproteins predicted by the same LipoP algorithm. Taken together, these findings may indicate the use of a different lipoprotein cleavage/synthesis mechanism by *M. suis*. Consequently, we are unable to correctly identify lipoproteins as they may have divergent lipoboxes. Given the presence of *secY* and *secA* genes in *M. suis*, these proteins are likely to be secreted through the Sec pathway. However, it is noteworthy that the signal peptidase I is also absent. This feature has been observed in other mycoplasmas and may further suggest these organisms have a different mechanism of protein cleavage [68].

Cytoplasmic and transmembrane proteases are crucial for survival and pathogenesis of bacteria. Transmembrane proteases may be used to acquire metabolic precursors from the host, inactivate host cell defense proteins, and participate in tissue degradation. However, only two transmembrane proteases of *M. suis* were detected: the metalloproteases CAAX amino terminal protease and ATP-dependent protease FtsH. CCAX amino terminal protease is involved in cleavage of the AAX tripeptide in the C-terminal of proteins and likely to be related to protein and/or peptide modification and secretion [69]. FtsH is a pleiotropic protein required for correct cell division in bacteria.

An o-sialoglycoprotein endopeptidase ortholog was detected in the *M. suis* genome. This enzyme, as described in *Mannheimia haemolytica*, may be involved in erythrocyte lysis, resulting from cleavage of *o*-sialoglycosylated proteins such as glycophorin A [70]. Although there is experimental evidence that this enzyme has extracellular activity in *M. haemolytica* [70], both PSORTb and SignalP algorithms predict it as cytoplasmic and non-secretory in mycoplasmas, including *M. suis*, and in *M. haemolytica*. The function of this protein in respect to erythrocyte lysis remains to be elucidated in mycoplasmas.

### Paralogous gene families

Many mycoplasmas have developed multi-gene families as a resource for antigenic diversity and means to avoid clearance by the host's immune system [71]. Variations in the number of paralogous genes in each family may also be related to increased virulence of specific bacterial strains [72], [73]. Sixty-nine paralogous gene families in *M. suis* comprising 365 CDSs (42.82%) were identified. The number of family members for each of the paralogs ranged from 2 to 37. This result is more than that reported in *M. penetrans*, in which 264/1038 (25.4%) of the predicted CDS were found in paralogous families [74]. While the large number of paralogs constitutes a plausible explanation for the large genome size of *M. penetrans* (1,358,633 bp), this isn't the case for *M. suis*, which is relatively small. All families, except for 8/69 (11.6%), were hypothetical proteins. The eight known families included 4 families of type I restriction system (n = 11, 6, 4 and 4) and 1 family each for the cobalt import ATP-binding protein CbiO (n = 2), DNA binding protein (n = 2), ATP synthase C subunit (n = 2), and Dna polymerase I (n = 2). Members of the four largest families (n = 37, 20, 16 and 13), all of which are hypothetical proteins, are clustered together in the genome (Figure 1), probably arising from in tandem duplication events. Additional evaluation of the ten largest paralogous gene families (n≥10) revealed that five of these families have probable transmembrane CDSs and 8 have secretory CDSs (with signal peptide sequence). Further studies should be conducted to evaluate the niche-specific pathogenesis-related function of these genes as well as their potential to provide antigenic diversity when infecting *M. suis* confronts the adaptive immune response of the pig.

### Conclusions

Our whole-genome analysis of *M. suis* provides new insights into its virulence and pathogenesis, antigenic variation, and gives us a better understanding of the nutrients and/or metabolites that are essential for its survival. While we were able to predict metabolic pathways, gaps remain to be filled and enzyme activities to be experimentally characterized. Nonetheless, the metabolic map developed herein will serve as the basis for future attempts to cultivate this organism *in vitro*.

Immunomodulatory mechanisms exerted by the *M. suis* during chronic infection are still unknown. The presence of large paralogous gene families should be further investigated in respect to antigenic variation and immune system evasion. The acute disease, however, may be exacerbated by a scavenging and competition mechanism for nutrients and/or metabolites. Overall, *M. suis* has a typical *Mollicutes* genome and is dependent on host cell metabolism for its survival (hemotrophic not just hemotropic), a characteristic that is likely to be linked to its pathogenicity.

## Materials and Methods

### Bacterial strain

The *M. suis* (*E. suis*) Illinois strain was obtained from Dr James Zachary, University of Illinois, Urbana-Champaign, IL [75]. This strain was initially isolated from a pig with acute bacteremia. The present study represents the second passage of this bacterium through experimental infection. Following the first passage of this strain, EDTA blood was harvested at peak bacteremia and stored in 1.0 mL aliquots at −80°C.

### Experimental infection

One 45-day old, female, mixed-breed pig obtained from the Purdue Swine Farm was used in this study. The animal was housed at the Purdue University Animal Care and Use Facilities and provided feed and water *ad libitum*. Blood samples that were collected on days 0 (arrival day at the housing facility), 4 and 7, were negative for *M. suis* infection using a species-specific real-time PCR (Guimaraes et al., unpublished). On day 7 the pig was splenectomized and 2 weeks later she was experimentally infected via intravenous injection of 1.0 ml of blood infected with the *M. suis* Illinois strain. Approximately 10 days later when a peak of bacteremia (>90% of red blood cells infected) occurred, blood samples were collected for *M. suis* DNA isolation.

Animal use and experimental infection were conducted under a protocol approved by the Purdue University Animal Care and Use Committee (Protocol #06-100). To control bacteremia and febrile episodes, the pig was treated at peaks of *M. suis* bacteremia with oxytetracycline (20 mg/kg, intramuscularly, twice daily) and flunixin meglumine (2.2 mg/kg, intramuscularly, once every 24 h) after blood collection.

### DNA extraction

A total of 30 mLs of blood were collected in EDTA containing tubes and promptly processed as previously described [75], [76]. Briefly, the blood was initially centrifuged for 5 min at 1,000× g. Plasma and buffy coat (white blood cells and platelets) were removed with a sterile Pasteur pipette. The red blood cells were resuspended in 3 volumes of 1× PBS-Tween and mixed by inversion for 4 to 8 hours to allow organisms to detach from the erythrocytes. Following centrifugation for 5 min at 1,000× g, the supernatant, which contains detached organisms, was harvested and filtered with 5.0 µm and 1.2 µm syringe-top filters to eliminate white blood cells. Organisms were then ultracentrifuged for 60 minutes at 22,000× g at 4°C in sterile tubes; the pellet resuspended in 1× PBS and extracted using a commercial kit following manufacturer's instructions (Quick-gDNA Miniprep, Zymo Research Corporation, Orange, CA). Quality and quantity of DNA was assessed by UV spectrophotometry (NanoDrop 2000, Thermo Scientific, Wilmington, DE, USA). The final quantity obtained as measured by Pico Green double stranded DNA fluorimetry was 18.7 µg of DNA; 5 µg of this extracted DNA were used for sequencing.

### DNA sequencing, sequence assembly, gap closure and validation

Whole-genome sequencing of *M. suis* was performed at the Purdue University's Genomics Core Facility using a GS-FLX (454) and Titanium chemistry to sequence a 3 kb paired end library. *Mycoplasma suis* genomic DNA was initially sheared into 3 kb fragments using the Hydroshear DNA Shearing Device (Genomic Solutions, Ann Arbor, Michigan, USA) to create fragments for paired end libraries. Libraries were then created following standard procedures from the Roche Paired End Library Preparation Method Manual. From a quarter PicoTitrePlate (PTP) GS-FLX run, a total of 55 million bases of sequence comprising 395,524 filter-pass sequence reads were generated with an average read length of 140 bp after removal of adaptor sequence and splitting of reads into pairs. After removal of adaptor sequences this came to roughly 55 million bases of sequence.

Reads were assembled using gs-Assembler (Version 2.3) (454 Life Sciences, Roche Applied Science). A total of 6 scaffolds resulted from this assembly; five of the shorter scaffolds, ranging in size from 3101 to 5614 bp, were identified as porcine DNA based on BLAST analysis (Sanger Institute and GenBank databases). The remaining scaffold (742,116 bp) did not show any significant BLAST hit to porcine sequences and was identified as *M. suis*. A mean genome coverage of 38.6 was obtained. A single gap that was located in this scaffold was closed with a primer walking approach and bidirectional sequencing by the Sanger method at Purdue University's Genomics Core Facility. The sequence data was submitted to the NCBI under accession number CP002525 (ADWK01000001). The genome sequence of *M. suis* was validated by comparison of virtual fingerprint patterns with that of fragments derived from pulsed-field gel electrophoresis (PFGE) of rare cutting restriction enzymes as previously reported [76]. In addition, a Lambda ZAPII library of *M. suis* was constructed; the sequence of 20 inserts (approximately 0.2 to 4.5 kb each) from immunoreactive clones (data not shown) in this library were compared to that of the *M. suis* genomic sequence.

### Identification of CDS and annotation

Automated gene prediction was done using Glimmer version 3.02 [77]. Briefly, an initial set of CDSs was identified using Glimmer (Genetic Code Table 4) and analyzed using the annotation engine Manatee from The Institute for Genomic Sciences (University of Maryland, School of Medicine, Baltimore, MD). CDSs showing a HMM (Hidden Markov Model) score above the trusted threshold, ≥45% identity and ≥90% coverage to known gene sequences according to BER (BLAST-Extended-Repraze) were selected. These sequences, in addition to long CDSs extracted using the Glimmer's “long-orfs” program, were subsequently used to train Glimmer 3.02 to recognize potential CDSs in the whole *M. suis* genome as a final prediction. DNA regions not contained in this final prediction set were extracted using the Glimmer's “uncovered” program and blasted (blastx) against non-redundant proteins database from GenBank to allow for identification of missing CDSs.

The CDSs were again analyzed with Manatee. Individual CDS were manually curated; evidence generated by the pipeline (incuding BER, HMMs, PROSITE matches, TMHMM and SignalP) were used to infer annotations. CDSs with HMM score below the trusted value and less than 40% identity or only local similarities to known protein sequences, were called hypothetical proteins. tRNAs were located using tRNA-scan-SE [78]. Lipoproteins and signal peptides were identified using LipoP and SignalP algorithms, respectively [79]–[82], and paralogous gene families were recognized using BLASTCLUST analysis (National Center for Biotechnology Information, NCBI, Bethesda, MD), with 30% sequence identity and 70% covered length thresholds. Comparative analyses with other bacterial genomes were performed based on genome annotations deposited in the NCBI Genome database.

## Supporting Information

Figure S1 Metabolic map of *Mycoplasma suis*.
(PDF)

Table S1 Abbreviations of the pathway metabolites.
(DOC)

Table S2 Abbreviations of pathway enzymes, their E.C., gene name/numbers and reaction(s) they catalyze.
(DOC)
