# Supplementary material for: Complete Genome Sequence of Mycoplasma suis and Insights into Its Biology and Adaption to an Erythrocyte Niche
Source: PLoS One. 2011 May 10;6(5):e19574. doi: 10.1371/journal.pone.0019574 (PMC3091866; doi:10.1371/journal.pone.0019574)
Supplement: Figure S1 — Metabolic map of Mycoplasma suis. (PDF) [file pone.0019574.s001.pdf]

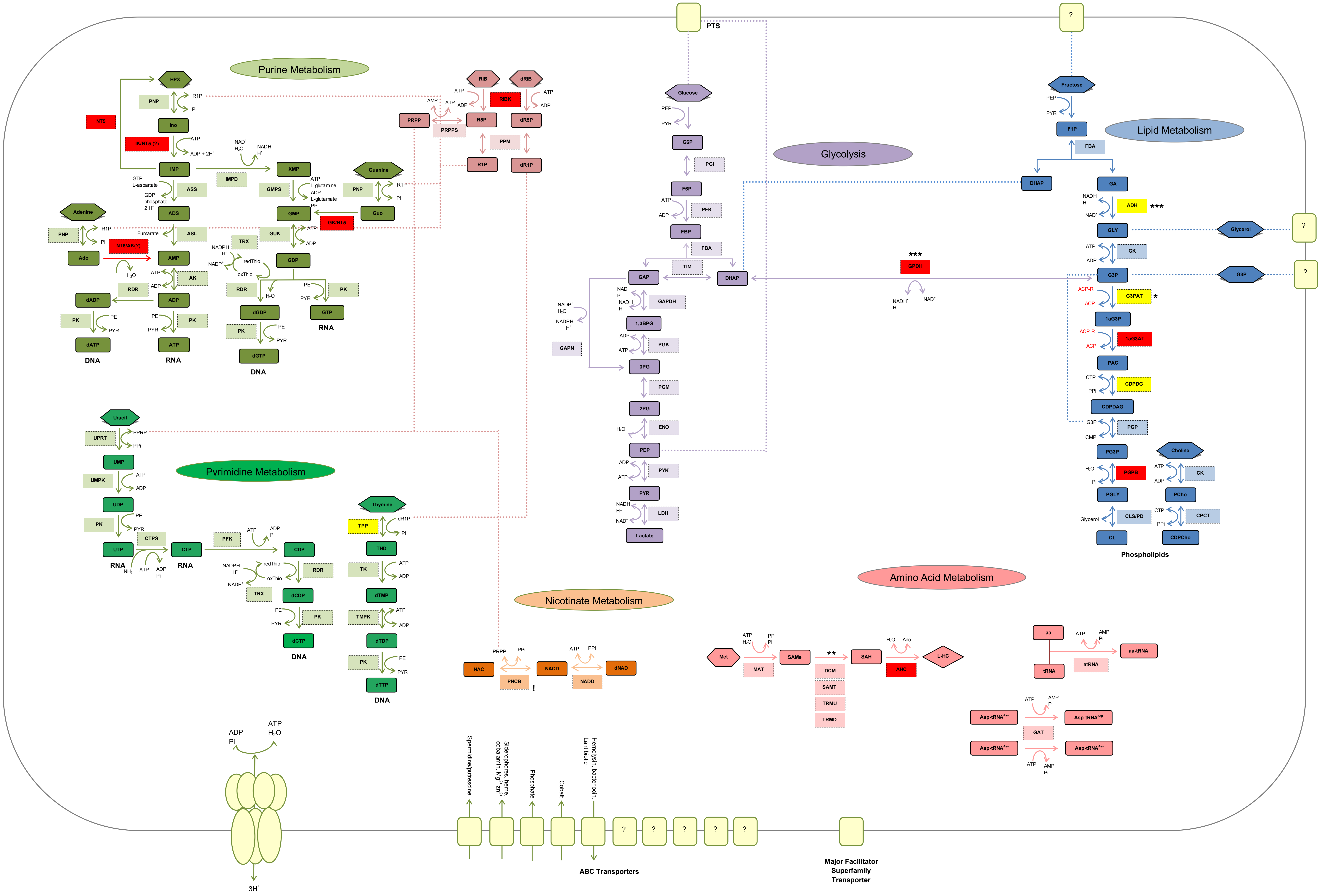

Figure S1. Metabolic map of *Mycoplasma suis*. Dashed boxes are enzymes. Yellow boxes represent enzymes with no orthologs in the genome but present in the proteomic study performed by Yuan et al [48]. Red boxes represent enzymes with no orthologs in the genome but likely to be present given the existence of others. Red arrows represent reactions in which the directionality is not established. Hexagons represent molecules that are uptaken by the microorganism, whereas diamonds represent exported metabolities. * = two proteins with acyl transferase properties were found in the study performed by Yuan et al. [48]; ** = different products are produced depending on the enzyme; *** = MSU_0539 has an alcohol/glycerol dehydrogenase domain (E-value 9.65 e-05) but only 65% coverage and 18% identity to known *Mollicutes* CDSs (NADH dependent butanol dehydrogenase from *M. penetrans*; E-value 4e-06). ! = presence of a frameshift mutation. MSU_0501 has a Blast hit with relatively high E-value (0.007) to CDPDG (Phosphatidate cytidylyltransferase) from *Streptococcus* sp.; experimental studies should be conducted to evaluate enzymatic activity of this protein.
