# Supplementary material for: Complete Genome Sequence of Mycoplasma suis and Insights into Its Biology and Adaption to an Erythrocyte Niche
Source: PLoS One. 2011 May 10;6(5):e19574. doi: 10.1371/journal.pone.0019574 (PMC3091866; doi:10.1371/journal.pone.0019574)
Supplement: Table S1 — Abbreviations of the pathway metabolites. (DOC) [file pone.0019574.s002.doc]

**Table S1.** Abbreviations of the pathway metabolites.

| **Abbr.** | **Metabolite** |
| --- | --- |
| 1,3BPG | D-glycerate 1,3-bisphosphate |
| 1aG3P | 1-acyl-sn-glycerol 3-phosphate |
| 2PG | 2-phospho-D-glycerate |
| 3PG | 3- phosphor-D-glycerate |
| Aa | Amino acid |
| aa-tRNA | aminoacyl‐tRNA |
| ACP | Acyl‐carrier‐protein, holo[acyl‐carrier‐protein] |
| ACP-R | Acyl‐[acyl‐carrier protein] |
| Ado | Adenosine |
| ADP | Adenosine 5’ diphosphate |
| ADS | Adenylosuccinate |
| AMP | Adenosine 5’ monophosphate |
| Asp | L-aspartate |
| ATP | Adenosine 5’ triphosphate |
| CDP | Cytidine 5’ diphosphate |
| CDPCho | CDP-choline |
| CDPDAG | CDP‐diacylglycerol |
| CL | Cardiolipin |
| CTP | Cytidine 5’ monophosphate |
| dADP | Deoxyadenosine 5’ diphosphate |
| dATP  dCDP | Deoxyadenosine 5’ triphosphate  Deoxycytidine 5’ diphosphate |
| dCTP | Deoxyxitidine 5’ triphosphate |
| dGDP | Deoxyguanosine 5’ diphosphate |
| dGTP | Deoxyguanosine 5’ triphosphate |
| DHAP | glycerone phosphate, dihydroxyacetone phosphate |
| dR1P | Deoxyribose 1’ phosphate |
| dR5P | Deoxyribose 5’ phosphate |
| dRIB | Deoxyribose |
| dTDP | Deoxythymidine 5’ diphosphate |
| dTMP | Deoxythymidine 5’ monophosphate |
| dTTP | Deoxythymidine 5’ triphosphate |
| F1P | Fructose 1’ phosphate |
| F6P | Fructose 6’ phosphate |
| **Abbr** | **Metabolite** |
| FBP | Fructose 1,6 biphosphate |
| G3P | Glycerol 3’ phosphate |
| G6P | Glucose 6’ phosphate |
| GA | Glyceraldehyde |
| GAP | D‐glyceraldehyde 3‐phosphate |
| GDP | Guanosine 5’ diphosphate |
| GLY | Glycerol |
| GMP | Guanosine 5’ monophosphate |
| GTP | Guanosine 5’ triphosphate |
| Guo  HPX | Guanosine  Hypoxanthine |
| IMP | Inosine 5’ monophosphate |
| Ino | Inosine |
| L-HC | L‐homocysteine |
| Met | Methionine |
| NAC | Nicotinate |
| NACD | Nicotinate D-ribonucleotide |
| dNAD | Deamino NAD^+^ |
| NAD+ | Nicotinamide adenine dinucleotide |
| NADH | Reduced nicotinamide adenine dinucleotide |
| NADP+ | Nicotinamide adenine dinucleotide phosphate |
| NADPH  oxThio | Reduced nicotinamide adenine dinucleotide phosphate  Thioredoxin disulfide |
| PAC | phosphatidate, 1,2‐diacyl‐sn‐glycerol 3‐phosphate |
| PCho | Choline phosphate |
| PEP | Phosphoenolpyruvic acid |
| PG3P | phosphatidylglycerophosphate, 3(3‐sn‐phosphatidyl)‐sn‐glycerol 1‐phosphate |
| PGLY | Phosphatidylglycerol |
| Pi | Phosphate |
| PPi | Diphosphate |
| PRPP | 5’ Phosphoribosyl 1’ pyrophosphate |
| PTS | Phosphotransferase system |
| PYR | Pyruvate |
| R1P | Ribose 1’ phosphate |
| **Abbr.** | **Metabolite** |
| R5P | Ribose 5’ phosphate |
| redThio | Thioredoxin |
| RIB | Ribose |
| SAH | S‐adenosyl‐L‐homocysteine |
| SAMe | S‐adenosyl‐L‐methionine |
| THD | Thymidylate |
| UDP | Uridine 5’ diphosphate |
| UMP | Uridine 5’ monophosphate |
| UTP | Uridine 5’ triphosphate |
| XMP | Xanthosine 5’ monophosphate |
