# Supplementary material for: Complete Genome Sequence of Mycoplasma suis and Insights into Its Biology and Adaption to an Erythrocyte Niche
Source: PLoS One. 2011 May 10;6(5):e19574. doi: 10.1371/journal.pone.0019574 (PMC3091866; doi:10.1371/journal.pone.0019574)
Supplement: Table S2 — Abbreviations of pathway enzymes, their E.C., gene name/numbers and reaction(s) they catalyze. (DOC) [file pone.0019574.s003.doc]

**Table S2.** Abbreviations of pathway enzymes, their E.C., gene name/numbers and reaction(s) they catalyze.

| **Abbr.** | **Enzyme name** | **E.C.** | **Gene name** | **Gene number** | **Reaction(s)** | **RN** |
| --- | --- | --- | --- | --- | --- | --- |
| **Purine Metabolism** | |  |  |  |  |  |
| AK | Adenylate kinase | 2.7.4.3 | adk | MSU_0439 | ATP + AMP = 2 ADP | R00127 |
| ASL | Adenylosuccinate lyase | 4.3.2.2 | purB | MSU_0708 | N6-(1,2-dicarboxyethyl) AMP = fumarate + AMP | R01083 |
| ASS | Adenylosuccinate synthase | 6.3.4.4 | purA | MSU_0292 | GTP + IMP + L-aspartate = GDP + phosphate + N6-(1,2-dicarboxyethyl)-AMP | R01135 |
| GK | Guanosine kinase | 2.7.1.73 | gsk | ? | ATP + Guanosine = ADP + GMP | R01228 |
| GMPS | Guanosine monophosphate synthase | 6.3.5.2 | guaA | MSU_0479 | ATP + xanthosine 5'-phosphate + L-glutamine + H2O = AMP + diphosphate + GMP + L-glutamate | R01231 |
| GUK | Guanylate kinase | 2.7.4.8 | gmk | MSU_0372 | ATP + GMP = ADP + GDP | R00332 |
| IK | Inosine kinase | 2.7.1.73 | gsk | ? | ATP + inosine = ADP + IMP | R01131 |
| IMPD | Inosine monophosphate dehydrogenase | 1.1.1.205 | guaB | MSU_0478 | 5'-phosphate + NAD+ + H2O = xanthosine 5'-phosphate + NADH + H+ | R01130 |
| NT5 | 5'‐nucleotidase | 3.1.3.5 | ushA | ? | 5'-ribonucleotide + H2O = a ribonucleoside + phosphate | R07297 |
| PK | Pyruvate kinase | 2.7.1.40 | pyk | MSU_0701 | phosphoenolpyruvate + dADP = pyruvate + dATP phosphoenolpyruvate + dGDP = pyruvate + dGTP phosphoenolpyruvate + ADP = pyruvate + ATP | R01138  R01858  R00200 |
| PNP | Purine‐nucleoside phosphorylase | 2.4.2.1 | deoD | MSU_0073 | adenosine + phosphate = adenine + D‐ribose 1‐phosphate |  |
| RDR | Ribonucleoside-diphosphate reductase | 1.17.4.1 | nrdA  nrdF | MSU_0309  MSU_0311 | 2'-deoxyribonucleoside diphosphate + thioredoxin disulfide + H2O = ribonucleoside diphosphate + thioredoxin | R04294 |
| TRX | Thioredoxin-disulfide reductase | 1.8.1.9 | trxB | MSU_0480 | thioredoxin + NADP+ = thioredoxin disulfide + NADPH + H+ | R02016 |
| **Abbr.** | **Enzyme name** | **E.C.** | **Gene name** | **Gene number** | **Reaction(s)** | **RN** |
| **Pyrimidine Metabolism** | |  |  |  |  |  |
| UPRT | Uracil phosphoribosyltransferase | 2.4.2.9 | upp | MSU_0875 | UMP + diphosphate = uracil + 5-phospho-alpha-D-ribose 1-diphosphate | R00966 |
| UMPK | Uridylate kinase | 2.7.4.22 | pyrH | MSU_0503 | ATP + UMP = ADP + UDP | R00158 |
| PK | Pyruvate kinase | 2.7.1.40 | pyk | MSU_0701 | phosphoenolpyruvate + UDP = pyruvate + UTP phosphoenolpyruvate + dCDP = pyruvate + dCTP phosphoenolpyruvate + dTDP = pyruvate + dTTP | R00659  R02320  R02320 |
| CTPS | CTP synthase | 6.3.4.2 | pyrG | MSU_0362 | ATP + UTP + NH3 = ADP + phosphate + CTP | R00571 |
| PFK | 6-Phosphofructokinase | 2.7.1.11 | pfk | MSU_0702 | CTP + D-Fructose 6-phosphate = CDP + D-Fructose 1,6-bisphosphate | R00767 |
| RDR | Ribonucleoside‐ diphosphate reductase | 1.17.4.1 | nrdA  nrdF | MSU_0309  MSU_0311 | 2'-deoxyribonucleoside diphosphate + thioredoxin disulfide + H2O = ribonucleoside diphosphate + thioredoxin | R04294 |
| TRX | Thioredoxin-disulfide reductase | 1.8.1.9 | trx | MSU_0480 | thioredoxin + NADP+ = thioredoxin disulfide + NADPH + H+ | R02016 |
| TPP | Thymidine phosphorylase | 2.4.2.4 | deoA | ? | Thymidine + Orthophosphate = Thymine + 2-Deoxy-D-ribose 1-phosphate | R01570 |
| TK | Thymidine kinase | 2.7.1.21 | tdk | MSU_0066 | ATP + thymidine = ADP + thymidine 5'-phosphate | R01567 |
| TMPK | Thymidylate kinase | 2.7.4.9 | tmk | MSU_0829 | ATP + dTMP = ADP + dTDP | R02094 |
| **Ribose Metabolism** | |  |  |  |  |  |
| RIBK | Ribokinase | 2.7.1.15 | rbsK | ? | ATP + D-ribose = ADP + D-ribose 5-phosphate | R01051 |
| PPM | Phosphoglucomutase | 5.4.2.2 | cpsG | MSU_0035 | alpha-D-Ribose 1-phosphate = D-Ribose 5-phosphate | R01057 |
| PRPPS | Ribose-phosphate diphosphokinase | 2.7.6.1 | prs | MSU_0015 | ATP + D-ribose 5-phosphate = AMP + PRPP | R01049 |
| **Abbr.** | **Enzyme name** | **E.C.** | **Gene name** | **Gene number** | **Reaction(s)** | **RN** |
| **Glycolysis** | |  |  |  |  |  |
| PGI | Glucose-6-phosphate isomerase | 5.3.1.9 | pgiB | MSU_0013 | D-glucose 6-phosphate = D-fructose 6-phosphate | R00771 |
| PFK | 6-phosphofructokinase | 2.7.1.11 | pfk | MSU_0702 | ATP + D-fructose 6-phosphate = ADP + D-fructose 1,6-bisphosphate | R00756 |
| FBA | Fructose biphosphate aldolase | 4.1.2.13 | fba | MSU_0475 | D-fructose 1,6-bisphosphate = glycerone phosphate + D-glyceraldehyde | R01068 |
| TIM | Triose phosphate isomerase | 5.3.1.1 | tim | MSU_0071 | D-glyceraldehyde 3-phosphate = glycerone phosphate | R01015 |
| GAPDH | Glyceraldehyde-3-phosphate dehydrogenase | 1.2.1.12 | gap | MSU_0835 | D-glyceraldehyde 3-phosphate + phosphate + NAD+ = 3-phospho-D-glyceroyl phosphate + NADH + H+ | R01061 |
| GAPN | NADP-dependent glyceraldehyde dehydrogenase | 1.2.1.9 | gapN | MSU_0029 | D-glyceraldehyde 3-phosphate + NADP+ + H2O = 3-phospho-D-glycerate + NADPH + 2 H+ | R01058 |
| PGK | Phosphoglycerate kinase | 2.7.2.3 | pgk | MSU_0072 | ATP + 3-phospho-D-glycerate = ADP + 3-phospho-D-glyceroyl phosphate | R01512 |
| PGM | Phosphoglycerate mutase | 5.4.2.1 | gpml | MSU_0072 | 2-phospho-D-glycerate = 3-phospho-D-glycerate | R01518 |
| ENO | Enolase | 4.2.1.11 | eno | MSU_0703 | 2-phospho-D-glycerate = phosphoenolpyruvate + H2O | R00658 |
| PYK | Pyruvate kinase | 2.7.1.40 | pyk | MSU_0701 | ADP + phosphoenolpyruvate = ATP + pyruvate | R00200 |
| LDH | L-lactate dehydrogenase | 1.1.1.27 | ldh | MSU_0299 | pyruvate + NADH + H+ = lactate + NAD+ | R00703 |
| **Lipid metabolism** | |  |  |  |  |  |
| FBA | Fructose biphosphate aldolase | 4.1.2.13 | fba | MSU_0475 | D-fructose 1,6-bisphosphate = glycerone phosphate + D-glyceraldehyde | R01068 |
| ADH | Alcohol dehydrogenase | 1.1.1.1 | adh | ? | glyceraldehyde + NADH + H+ = glycerol + NAD+ | R01041 |
| **Abbr.** | **Enzyme name** | **E.C.** | **Gene name** | **Gene number** | **Reaction(s)** | **RN** |
| **Lipid Metabolism** | |  |  |  |  |  |
| G3PAT | Glycerol-3-phosphate O-acyltransferase | 2.3.1.15 | plsB | ? | acyl-CoA + sn-glycerol 3-phosphate = CoA + 1-acyl-sn-glycerol 3-phosphate | R00851 |
| 1aG3AT | 1-acyl-sn-glycerol-3-phosphate acyltransferase | 2.3.1.51 | plsC | ? | acyl-CoA + 1-acyl-sn-glycerol 3-phosphate = CoA + 1,2-diacyl-sn-glycerol 3-phosphate | R02241 |
| GK | Glycerol kinase | 2.7.1.30 | glpk | MSU_0290 | glycerol + ATP = ADP + glycerol 3‐phosphate | R00847 |
| CDPDG | Phosphatidate cytidylyltransferase | 2.7.7.41 | cdsA2 | MSU_0501? | CTP + phosphatidate = diphosphate + CDP-diacylglycerol | R01799 |
| PGP | CDP-diacylglycerol--glycerol-3-phosphate 3-phosphatidyltransferase | 2.7.8.5 | pgsA | MSU_0466 | CDP-diacylglycerol + sn-glycerol 3-phosphate = CMP + 3(3-sn-phosphatidyl)-sn-glycerol 1-phosphate | R01801 |
| PGPB | Phosphatidyl glycerophosphatase | 3.1.3.27 | pgpA | ? | phosphatidylglycerophosphate + H2O = phosphatidylglycerol + phosphate | R02029 |
| CLS/PD | Cardiolipin synthase | 2.7.8.- | cls | MSU_0014 | 2 Phosphatidylglycerol = Cardiolipin + Glycerol | R07390 |
| CK | Choline kinase | 2.7.1.32 | licA | MSU_0492 | ATP + choline = ADP + O-phosphocholine | R01021 |
| CPCT | Choline-phosphate cytidylyltransferase | 2.7.7.15 | pct | MSU_0351 (?) | CTP + choline phosphate = diphosphate + CDP-choline | R01890 |
| GPDH | Glycerol‐3‐phosphate dehydrogenase | 1.1.99.5 | gpsA | MSU_0539 (?) | glycerol 3‐phosphate + NAD+ = glycerone phosphate + NADH + H+ |  |
| **Nicotinate metabolism** | |  |  |  |  |  |
| PNCB | Nicotinate phosphorybosyltransferase | 2.4.2.11 | pncB | MSU_0347  MSU_0348 | nicotinate + 5-phospho-alpha-D-ribose 1-diphosphate = nicotinate D-ribonucleotide + diphosphate | R01724 |
| NADD | Nicotinate-nucleotide adenylyltransferase | 2.7.7.18 | nadD | MSU_0351 | ATP + nicotinate ribonucleotide = diphosphate + deamido-NAD+ | R03005 |
| **Abbr.** | **Enzyme name** | **E.C.** | **Gene name** | **Gene number** | **Reaction(s)** | **RN** |
| **Amino acid metabolism** | |  |  |  |  |  |
| MAT | methionine adenosyltransferase | 2.5.1.6 | metK | MSU_0082 | ATP + L-methionine + H_2_O = phosphate + PPi +SAM | R00177 |
| DCM | DNA (cytosine‐5‐)‐ methyltransferase | 2.1.1.37 | hpaIIM | MSU_0397 | SAM + DNA = S-adenosyl-L-homocysteine + DNA containing 5-methylcytosine | R00380 |
| SAMT | S-adenosyl-L-methionine-dependent methyltransferase | 2.1.1.12 | mraW | MSU_0409 | S-adenosyl-L-methionine + L-methionine = S-adenosyl-L-homocysteine + S-methyl-L-methionine | R00649 |
| TRMD | tRNA (guanine-N(7)-)-methyltransferase | 2.1.1.33 | trmD | MSU_0450 | S-adenosyl-L-methionine + tRNA = S-adenosyl-L-homocysteine + tRNA containing N7-methylguanine | R00600 |
| TRMU | tRNA (5-methylaminomethyl-2-thiouridylate)-methyltransferase | 2.1.1.61 | trmU | MSU_0305 | S-adenosyl-L-methionine + tRNA = S-adenosyl-L-homocysteine + tRNA containing 5-methylaminomethyl-2-thiouridylate | R00601 |
| AHC | adenosylhomocysteinase | 3.3.1.1 | ahcy | ? | S-adenosyl-L-homocysteine + H2O = L-homocysteine + adenosine | R00192 |
| GAT | aspartyl/glutamyl-tRNA(Asn/Gln) amidotransferase | 6.3.5.6  6.3.5.7 | gatA  gatB  gatC | MSU_0487  MSU_0787  MSU_0486 | ATP + L-glutamyl-tRNA(Gln/Asn) + L-glutamine = ADP + phosphate + L-glutaminyl-tRNA(Gln/Asn) + L-glutamate | R04212  R03905 |
| atRNA | Aminoacyl-tRNA synthases | 6.1.1.- |  | Several genes | ATP + amino acid + tRNA = AMP + diphosphate + aminoacyl-tRNA |  |
